# Nanoscale manipulation of the Mott insulating state coupled to charge order in 1*T*-TaS_2_

**DOI:** 10.1038/ncomms10453

**Published:** 2016-01-22

**Authors:** Doohee Cho, Sangmo Cheon, Ki-Seok Kim, Sung-Hoon Lee, Yong-Heum Cho, Sang-Wook Cheong, Han Woong Yeom

**Affiliations:** 1 Center for Artificial Low Dimensional Electronic Systems, Institute for Basic Science (IBS), 77 Cheongam-Ro, Pohang 790-784, Korea; 2 Department of Physics, Pohang University of Science and Technology (POSTECH), Pohang 790-784, Korea; 3 Laboratory for Pohang Emergent Materials and Max Planck POSTECH Center for Complex Phase Materials, Pohang University of Science and Technology, Pohang 790-784, Korea; 4 Rutgers Center for Emergent Materials and Department of Physics and Astronomy, Rutgers University, Piscataway, New Jersey 08854, USA

## Abstract

The controllability over strongly correlated electronic states promises unique electronic devices. A recent example is an optically induced ultrafast switching device based on the transition between the correlated Mott insulating state and a metallic state of a transition metal dichalcogenide 1*T*-TaS_2_. However, the electronic switching has been challenging and the nature of the transition has been veiled. Here we demonstrate the nanoscale electronic manipulation of the Mott state of 1*T*-TaS_2_. The voltage pulse from a scanning tunnelling microscope switches the insulating phase locally into a metallic phase with irregularly textured domain walls in the charge density wave order inherent to this Mott state. The metallic state is revealed as a correlated phase, which is induced by the moderate reduction of electron correlation due to the charge density wave decoherence.

## Abstract (web summary)

Controlled switching between states in materials with strong electronic correlations can form the basis for functional devices. Here, the authors use a scanning tunnelling microscope tip to switch between a Mott insulator state and a metallic state with charge-density-wave domains in 1*T*-TaS_2_.

## Graphical abstract

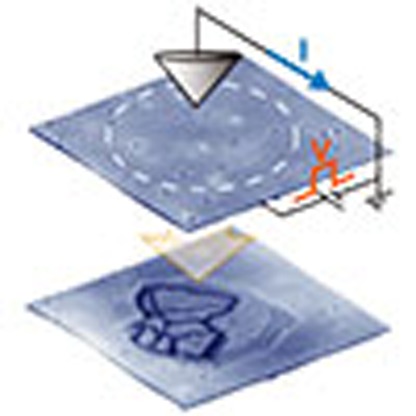

The electron motion as represented by the bandwidth *W* is strongly limited by the on-site Coulomb repulsion *U* and a Mott insulating state develops by the localization of electrons near the Fermi energy (*E*_F_) when *U*/*W* exceeds a critical value^1^,^2^,^3^,^4^. In the Mott insulator 1*T*-TaS_2_, a unique correlated insulating state is brought on by the spontaneous formation of the charge density wave (CDW) order, which substantially reduces (increases) the bandwidth *W* (*U*/*W*) at *E*_F_ (refs 5, 6). Thus, the Mott phase becomes metallic upon increasing the temperature above *T*_c_∼220 K (ref. 7), where the CDW order melts into small domains textured by nearly commensurate domain wall networks^8^. The metallic phase with the textured CDW can be generated not only by thermal excitation but also by chemical doping^9^,^10^,^11^, photoexcitation^12^,^13^, pressure^14^, carrier injection^15^,^16^ and the reduction of thickness^17^,^18^.

While these studies demonstrate the macroscopic controllability of the correlated Mott insulating phase by the CDW order, the origin of the excited metallic phase, the textured CDW phase, has been elusive. The metallicity was attributed to the metallic domain wall themselves^14^, the metallization of the Mott-CDW domains due to the screening by free carriers of domain walls^9^, or the change of the interlayer stacking order^19^. However, there has been no experimental verification of these scenarios and no direct information on the electronic structure of the domain wall. The nature of the metallic phase is also in the centre of the current debate on the mechanism of superconductivity emerging at low temperatures^10^,^11^,^14^,^20^.

In the present study, we have succeeded in the nanoscale manipulation of the metal–insulator transition of the Mott insulating phase of 1*T*-TaS_2_. The metallic domains can reversibly be formed and erased with an atomically abrupt phase boundary by applying voltage pulse from a scanning tunnelling microscope (STM) tip. The spectroscopic measurements with atomic resolution rule out the existence of substantial free carriers along domain walls and unveil the correlated nature of the metallic phase.

## Result

### Phase manipulations of the charge ordered Mott insulator

**Figure 1:**
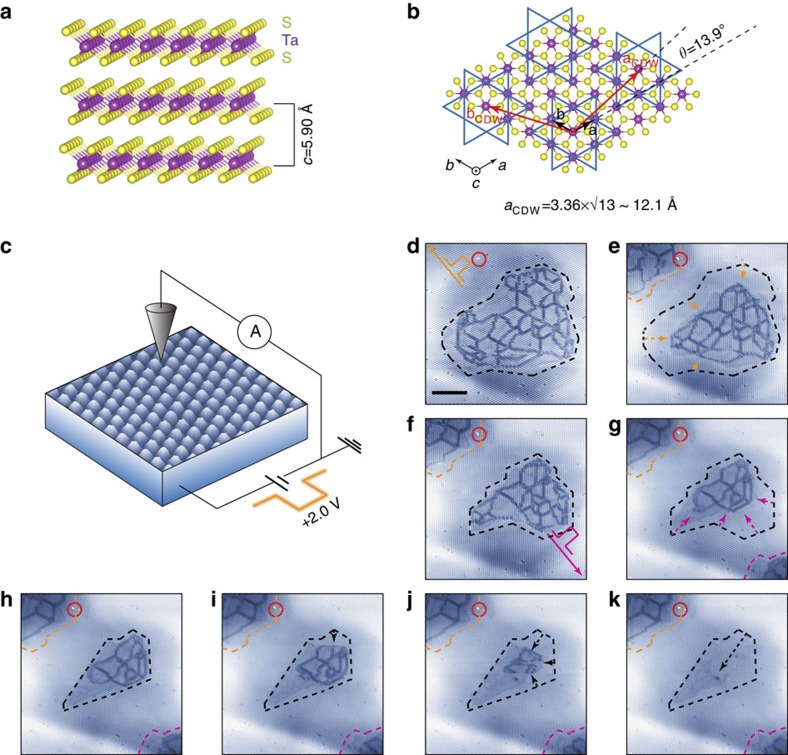
Nanoscale manipulations via positive voltage pulses to the CDW domain. (**a**) Side and (**b**) top view of the atomic structure of 1*T*-TaS_2_ with the David-star CDW pattern (blue lines) superimposed. Ta (S) atoms are displayed by purple (yellow) balls. The black (red) arrows indicate the lattice vectors of 1 × 1 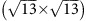 structure. (**c**) STM set-up with an image of the CDW phase (tunnelling current *I*_t_=100 pA, sample bias *V*_s_=−0.80 V and scan size *L*^2^=12 × 12 nm^2^) of 1*T*-TaS_2_ at *T*=4.3 K. (**d**–**k**) A series of STM images (*I*_t_=100 pA, *V*_s_=−1.20 V and *L*^2^=92 × 92 nm^2^) showing the time evolution of domains with the broken phase coherence of the CDW order by the domain wall network. An impurity (red circles) is used as a landmark for all images. Before the STM images of (**d**,**e**,**g**) voltage pulses are applied with an amplitude of +2.0, +2.10 and +2.35 V with a duration of 100 ms at the central, upper-left (orange arrow in **d**) and lower-right (magenta arrow in **f**) sites, respectively. The dashed lines and arrows indicate the pulse-induced evolution of domains. Scale bar, 20 nm.

Figure 1a illustrates the CdI_2_-type crystal structure of 1*T*-TaS_2_ with Ta atoms octahedrally coordinated by S atoms. A unit layer consists of one Ta layer sandwiched between two S layers. Within the insulating phase at low temperature, 1*T*-TaS_2_ develops a long-range ordered CDW accompanied with the David-star distortion; 12 Ta atoms shrink towards the centre Ta atom and S layers swell up along the *c* axis. Such a deformation forms a commensurate 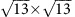 triangular superlattice (*a*_CDW_∼12.1 Å; Fig. 1b)^21^. The lattice deformation brings about the charge localization at the centre of David-star, which is clearly resolved in the STM image of the CDW phase (Fig. 1c)^22^.

The manipulation of the Mott-CDW phase was realized by applying a positive voltage pulse (*V*_s_≥+2.0 V and Δ*t*=100 ms) within a typical STM set-up at *T*=4.3 K (Fig. 1c; Methods). A pulse creates a textured CDW domain of a few tens of nanometres with an irregular domain wall network inside. The additional pulse nearby (solid arrows in Fig. 1d,f) can also reduce the size of a pre-existing textured CDW domain (dashed arrows in Fig. 1e,g). The higher a pulse voltage is, the bigger textured domain is formed. At this temperature, the large textured CDW domains are rather stable but the smaller one can be gradually reduced by the STM imaging with a moderate tunnelling current (Fig. 1h–k). This indicates the metastability of the textured CDW phase and that we can reproducibly induce a textured CDW domain at a desired position and erase it (Supplementary Fig. 1).

It has been shown that the Mott-CDW phase can be macroscopically turned into the metallic phase with textured CDW by thermal excitation^7^ or carrier doping^15^,^16^,^17^,^18^. They commonly introduced the crucial role of extra carriers and, in particular, the temperature-dependent Hall measurement^23^ and the electric-field-effect study^16^ indicated the direct role of hole carriers over the critical density in creating domain walls. In the present case, only a positive V-pulse is active, where the local hole concentration under the STM tip is enhanced^24^. This is consistent with the hole-carrier mechanism, while we cannot completely exclude the local heating effect and the transient electron carrier injection by the tunnelling current.

### Electronic structure of the domain walls

**Figure 2:**
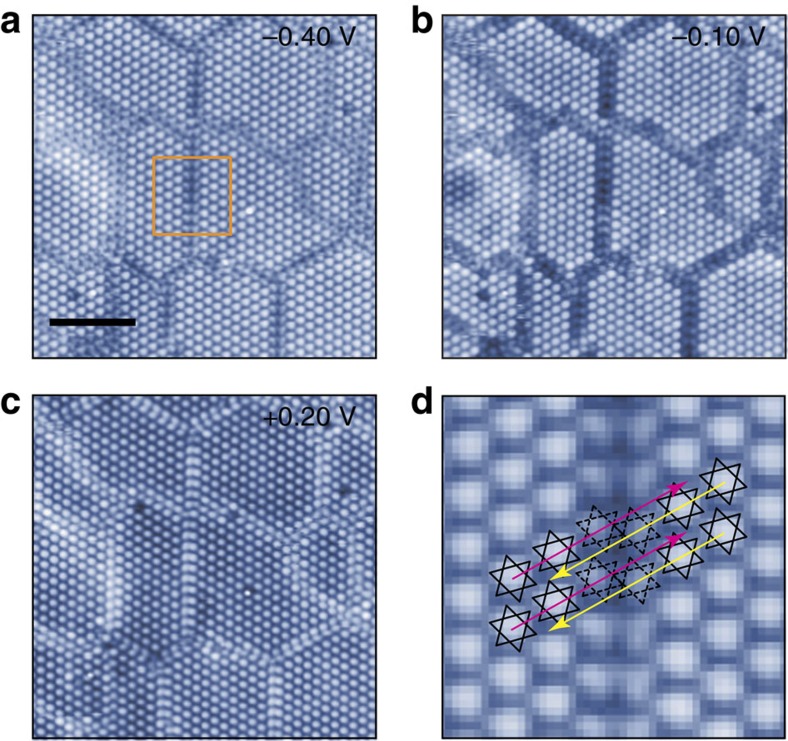
STM images of the textured CDW phase with domain walls. (**a**–**c**) Bias-dependent STM images showing the structure and electronic states of domain walls in the textured CDW phase (*I*_t_=100 pA, *V*_s_=−0.40, −0.10 and + 0.20 V and *L*^2^=40 × 40 nm^2^). Scale bar, 10 nm. (**d**) Zoomed-in of the orange box in **a**. The CDW direction of left (right) domain is marked by magenta (yellow) arrows to highlight the CDW phase shift across the domain wall. The dashed David-stars cannot construct perfect CDW unit cells due to the misfit at the domain wall.

The domain walls themselves are well resolved in the STM images (Fig. 2). The phase of the CDW changes abruptly across the domain walls as shown in Fig. 2d. They form disordered networks in contrast to the hexagonally ordered domain walls of the nearly commensurate CDW phase^8^,^25^. This difference is apparently related to the quenched nature of the present textured CDW domains, being far away from the thermal equilibrium. The present case would be closer to the metallic phase induced by the laser excitation from the low temperature phase. This work called the metallic phase a hidden, thermodynamically unreachable, order state and assumed a triangular lattice of the domain walls without any microscopic information^13^.

**Figure 3:**
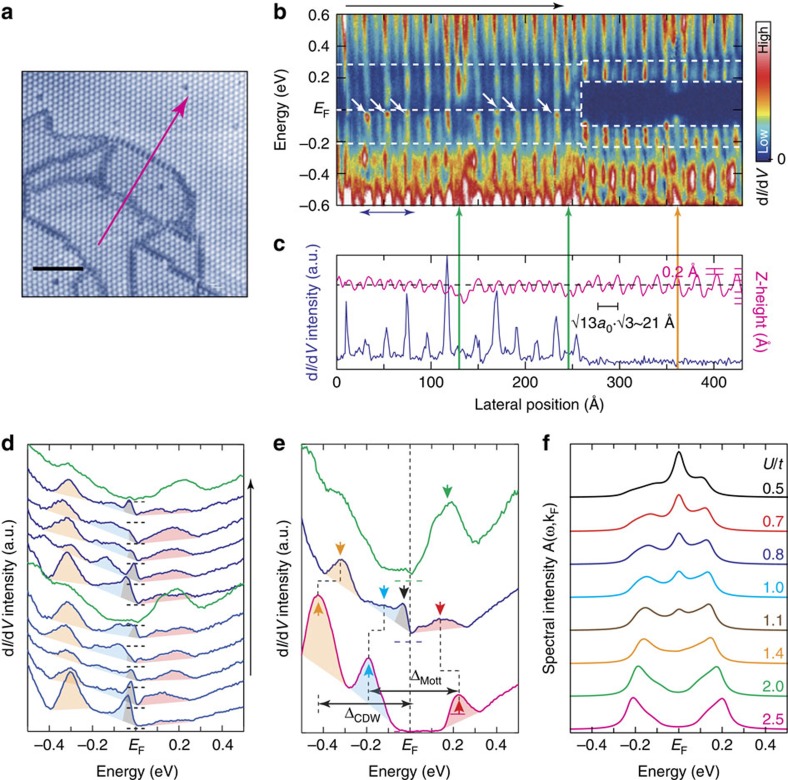
Direct comparison of electronic structures between commensurate and textured CDW phases. (**a**) STM image of a crossover region between the commensurate and textured CDW phase (*I*_t_=10 pA, *V*_s_=−1.20 V and *L*^2^=45 × 45 nm^2^). Scale bar, 10 nm. (**b**) The differential conductance d*I*/d*V*(*r*, *V*) data measured along the arrow in **a**. The white dashed lines and arrows highlight the bandwidth broadening and the coherent peaks, respectively. (**c**) The magenta and blue curve each correspond to the STM topographic line profile (Z-height) and the d*I*/d*V* intensity at *E*_F_ along the given lateral positions. The domain walls (a point defect) are marked by green(orange) arrows. (**d**) d*I*/d*V*(*V*) curves along the black arrow shown in **b**. Each curve is averaged within each CDW cluster and shifted for comparison. The spectra in green are for the domain walls in the textured CDW region. Coloured regions highlight the prominent peaks indicated by arrows in **e**. (**e**) Spatially averaged d*I*/d*V*(*V*) curves of the insulating commensurate CDW phase (magenta), the metallic domain (blue) and the domain wall (green). The horizontal dashed lines mark the zero conductance. Blue (red), yellow and green arrows indicate the lower (upper) Hubbard state, the topmost valence subband, and the domain wall state, respectively. (**f**) Theoretically calculated spectral function for different correlation *U*/*t* in a triangular lattice.

Most of the previous studies considered the conducting channel along a domain wall as the origin of the metallic property^9^,^14^. Nevertheless, there has been a lack of direct spectroscopic information on domain walls. The present study rules out a dominating metallic nature of the domain wall denying most of the previous metallization scenarios for various textured CDW phases. In the STM image, the contrast of domain walls is suppressed close to *E*_F_ (Fig. 2b) and enhanced at *V*_*s*_=+0.20 V (Fig. 2c) with their own reconstruction different from a David-star. This indicates that a domain wall has little density of states near *E*_F_ with its own electronic state at +0.20 eV, which is more directly verified by the spatially resolved d*I*/d*V*(*r*, *V*) curves (green curves in Fig. 3d,e). In addition, as shown in Fig. 2d, the common domain walls consist of even number (12+12) Ta atoms so that the number of electrons within the domain wall is even. Thus, the metallic character of the domain wall^26^ is not expected *a priori*. This point will be detailed elsewhere.

### Electronic structure of the textured CDW domain

In stark contrast, the textured CDW domain has a metallic characteristic. We took the d*I*/d*V*(*r*, *V*) curves crossing a boundary between a textured and a ordered CDW domain (Fig. 3). In the ordered CDW region, two prominent peaks at −0.19 and +0.23 eV are resolved, which correspond to the lower and upper Hubbard bands, respectively (Fig. 3b and the bottom curve in Fig. 3e). They have a bandwidth of ∼100 meV and constitute the Mott gap of ∼0.42 eV. Beyond the Mott gap, there is additional band splitting away from *E*_F_ around −0.30 eV. This bandgap is known to come from the CDW formation^6^ and the peak at −0.42 eV is ascribed to the top of the valence subbands^9^,^27^,^28^,^29^. This band splitting makes the necessary condition for the Mott insulating state, that is, a narrow band at *E*_F_.

Within the textured CDW domain, the tunnelling spectra unambiguously indicate finite density of states around *E*_F_. The zero-bias conductance profile (d*I*/d*V*(*r*, 0)) in Fig. 3c distinguishes the metallic and insulating regions sharply. Note that it is peaked on the CDW maxima within the textured CDW domain. They originate from a pronounced spectral feature close to *E*_F_ (white arrows in Fig. 3b, the grey regions in Fig. 3d and the black arrow in Fig. 3e). In addition, the tunnelling spectra in the textured CDW phase exhibit broad features centred at −0.12 and +0.14 eV. They are similar to the Hubbard states in the Mott-CDW phase but shifted slightly towards *E*_F_ with a substantial reduction of the intensity and a noticeable broadening (1.3–1.6 times larger than the bandwidth of Hubbard states). On the other hand, the top of the valence subband is also shifted substantially towards *E*_F_ (orange arrows in Fig. 3e).

### Calculations of the bandwidth-controlled Mott transition

The spectral characteristics of the textured CDW phase within the Mott gap can be directly related to a breakdown process of the Mott insulating state. The present system has been effectively described by a one-band Hubbard model on a triangular lattice at half filling. Our theoretical calculations based on spin-liquid physics^30^ (Methods) reveals a metal–insulator transition as a function of *U*/*t* (*t*, an intersite hopping integral proportional to *W*), which captures the major experimental findings; the weakening and broadening of the Hubbard states together with the reduction of the Mott gap and the appearance of the sharp peak near *E*_F_ (Fig. 3d). This peak was assigned as the coherent resonance of correlated electrons in the previous dynamic mean field theory calculations^31^. These theories assure that the present textured CDW phase is a correlated metallic state close to the critical regime of the Mott transition (*U*/*t*∼1.4 in Fig. 3f and Supplementary Fig. 2). Deviating marginally from the theory, the coherent peak assigned in the experiment has an asymmetric shape and is located slightly below *E*_F_. The previous STS results of a correlated metal showed very consistent spectra^32^. While we attribute those deviations to the interference effect caused by the tunnelling current^33^,^34^,^35^, a further study on the spectral details is desirable.

## Discussion

What remains to be explained is the origin of the reduced correlation *U*/*t* to drive the transition. The screening by free carriers of domain walls^9^ is not likely as shown by the lack of *E*_F_ weight in the d*I*/d*V* data. Instead, we note that the long-range CDW order is lost within the textured CDW domain. The reduced CDW order would naturally decrease the CDW band splitting, which is clearly evidenced by the substantial energy shift of the valence subband top (orange arrows in Fig. 3e). The effect of the reduced CDW bandgap on the Mott state can be traced by calculating the evolution of the bandwidth at *E*_F_. Our calculations unambiguously show that the width of the subband straddling the Fermi level at *U*=0 is linearly increased by the decrease of the CDW order parameter (Supplementary Fig. 3). The bandwidth increase of the Hubbard bands is evident in the experiment (Fig. 3). This is close to the concept of the bandwidth-controlled Mott transition discussed previously^14^,^36^. In addition, the domain walls can act as the disorder potential, which can also drive the metallization of a Mott insulator. The spatial fluctuation of the spectra within the textured CDW domain (Fig. 3d) seems to be related to the disorder^37^. However, this case yields broad spectral features with a symmetric dip at *E*_F_ (refs 38, 39), which is clearly distinguished from the sharp spectral feature close to *E*_F_ of the present case. On the other hand, the disorder is also known to reduce the correlation energy *U* in general^37^,^39^. This type of the disorder contribution is not excluded. Thus, we conclude that the reduced CDW order induces the increased bandwidth *W* and at the same time the reduced *U*, which drives the Mott-CDW state into the critical regime of a correlated metallic state.

**Figure 4:**
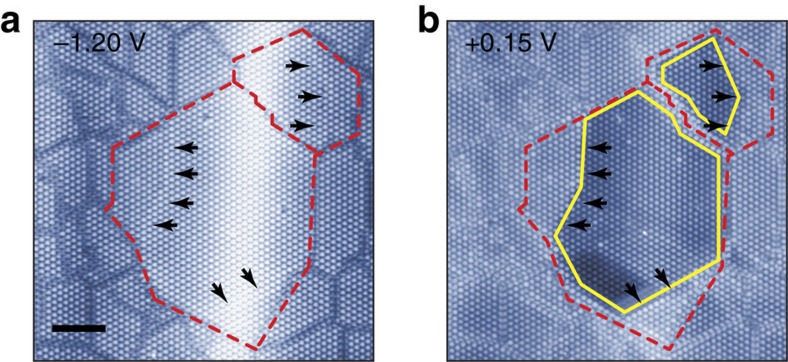
Existence of the subdomains and the stacking order. (**a**) High- and (**b**) low-bias STM image of the textured CDW domain (*I*_t_=10 pA, *V*_s_=−1.20 and +0.15 V and *L*^2^=62 × 62 nm^2^). The relatively large CDW domains (red dashed lines) within a texture CDW domain and the insulating subdomains (yellow solid lines) within them. The black arrows indicate the domain walls in the sublayers. Scale bar, 10 nm.

While the STM images we showed so far only deal with the lateral CDW ordering, we also notice that the vertical CDW stacking order has a crucial impact on this transition. Figure 4 shows that a relatively large domain within the metallic domain has extra but weak domain walls inside (black arrows in Fig. 4a), which is assigned as the domain wall existing in the sublayer(s). The low-bias images and the spectroscopy data indicate clearly that this domain harbours insulating subdomains as defined by the sublayer domain walls. One can straightforwardly deduce that these subdomains correspond to different interlayer stacking of the CDW; the interlayer CDW stacking order is lost by the formation of the irregular domain wall networks in each layer except for those metallic subdomains. This suggests that not only the intralayer but also interlayer CDW order has to be taken into account to explain the reduced electron correlation for the Mott transition^26^. The importance of the interlayer CDW stacking order was recently discussed, while the direct experimental information has been lacking^19^.

The present case of 1*T*-TaS_2_ is an exceptional example of the nanoscale control over strongly correlated electronic states. We can find only one case for the nanoscale manipulation of a Mott insulator in GaTa_3_Se_8_ (ref. 40). In this case, the strong electric-field-induced deformation of the lattice occurs and the transition is close to the avalanche dielectric breakdown^41^. In the present system, the transition is largely electronic and the resulting metallic state is a novel correlated state near the Mott criticality. The uniqueness of the present system lies on the intercoupled nature of the Mott state with the CDW order, which provides the extra tunability of the Mott state by a distinct order. The ultrafast switching capability of the present system^13^,^15^,^16^ in combination with the nanoscale controllability is expected to provide a unprecedented novel device platform based on correlated electronic systems.

## Methods

### Preparation of single crystal 1*T*-TaS_2_

The single crystal 1*T*-TaS_2_ were grown by iodine vapour transport method in the evacuated quartz tube. Before growth of the sample, the powder 1*T*-TaS_2_ was sintered for 48 h at 750 °C. We repeated this process two times to get poly crystals. To get high-quality sample, the seeds were slowly transported by iodine at 900–970 °C for 2 weeks. The tube was rapidly cooled down to room temperature in the air due to the metastability of the 1*T* phase.

### STM and STS measurements

The STM and STS measurements have been performed with a commercial STM (SPECS) in ultra high vacuum at *T*=4.3 K. The STM tips were prepared by mechanically sharpened Pt–Ir wires. All of STM images are acquired with the constant current mode with bias voltage *V*_s_ applied to the sample. When the positive *V*-pulses are applied to the sample with a fixed duration of *t*=100 ms and a varying amplitude, the feedback loop is opened with scanning condition (*V_s_*=−1.20 V and *I*_t_=100 pA). We can get the small texture CDW domain below *V_p_*∼+2.0 V and a larger textured CDW phase with increasing amplitude. The d*I*/*dV*(*V*) curves are recorded using a lock-in technique with voltage modulation *V*_*m*_=10 mV and frequency *f*=1 kHz.

### Theoretical calculations

The CDW-Mott state in 1*T*-TaS_2_ can be described by an effective one-band Hubbard model on the triangular lattice at half filling


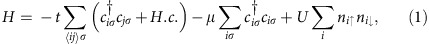


where *c*_*i*,*σ*_ is an electron annihilation operator with spin *σ* at site *i*, identified with each centre of David-star. Hinted from the observation that the coherent peak emerges in the textured CDW state, we take the U(1) slave-rotor representation for possible spin-liquid physics at least in the intermediate temperature regime^42^. It is straightforward to perform the saddle-point analysis based on an ansatz for spin-liquid physics^30^, which leads us to confirm the existence of a metal–insulator transition from a spin-liquid-type Mott insulating state to a correlated metallic phase at critical value of *U*/*t* (details shown in Supplementary Note 1).

## Additional information

**How to cite this article:** Cho, D. *et al*. Nanoscale manipulation of the Mott insulating state coupled to charge order in 1*T*-TaS_2_. *Nat. Commun.* 7:10453 doi: 10.1038/ncomms10453 (2016).

## Supplementary Material

Supplementary Information Supplementary Figures 1-3, Supplementary Note 1 and Supplementary References
