# Supplementary material for: Nanoscale manipulation of the Mott insulating state coupled to charge order in 1T-TaS2
Source: Nat Commun. 2016 Jan 22;7:10453. doi: 10.1038/ncomms10453 (PMC4735893; doi:10.1038/ncomms10453)
Supplement: Supplementary Information — Supplementary Figures 1-3, Supplementary Note 1 and Supplementary References [file ncomms10453-s1.pdf]

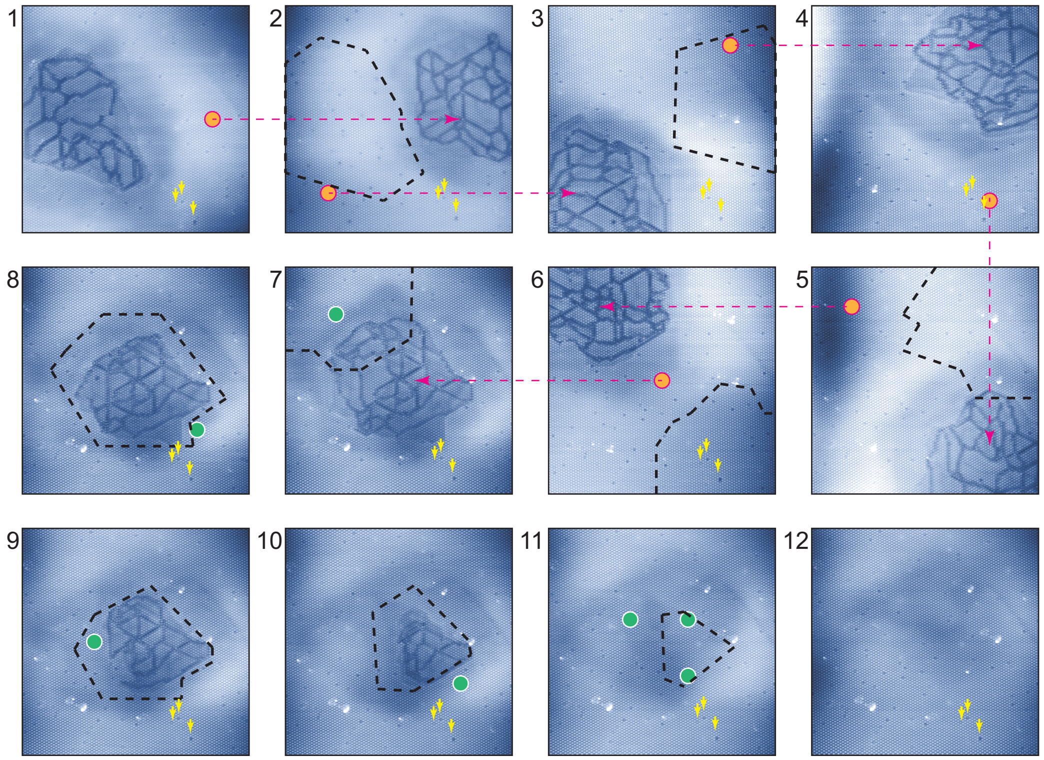

Supplementary Figure 1. **Creation and annihilation of the textured CDW domains.** A series of STM images of the V-pulse induced textured CDW domains ($I_t = 100$ pA, $V_s = -1.20$ V, $L^2 = 100 \times 100$ nm^2) including intrinsic several defects (a land mark indicated by yellow arrows). The black dashed lines mark the preexisting textured domain. The numbers present the sequence of manipulations. The voltage amplitude and pulse duration are fixed to +1.50 V and 100 ms. The field strength and the current amplitude are modulated by varying the tip height (z) with voltage pulses. We varied z values from -3 Å to -4 Å for weak (green dots) and strong (orange dots) pulses, respectively. The z-value indicates the variation of the tip position after the feedback loop is opened for the voltage pulses. Even though the voltage amplitude is lower than +2.0 V, the textured domain can be generated by approaching the STM tip to the sample. The strong pulses can not erase the textured domain without creation of new one. However, the weak pulses can be used only for annihilating the textured domain. The hidden states generated by macroscopic perturbations in $1T$-TaS_2 have been known to be annihilated by the thermal annealing process ($T \sim 70$ K) due to its prominent stability[1].

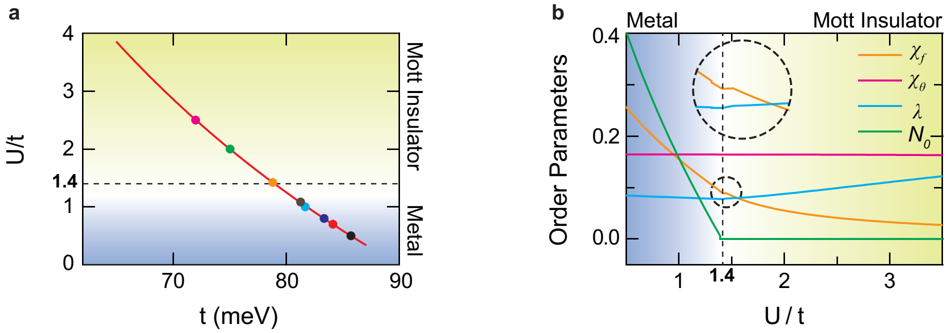

Supplementary Figure 2. ***U/t* controlled metal-insulator phase transition in spin-liquid description.** **(a)** The phase diagram and the way to control U and t for spectral functions in Fig. 3f. Each spectral function was acquired at the colored dots. **(b)** Using the parameters in **a**, the amplitude of order parameters, band renormalization (χ_f), width of incoherent band(χ_θ), Lagrange multiplier (λ), and coherent peak intensity (N_0) are calculated self-consistently. These parameters show the Mott transition at $U/t \approx 1.4$. The circle-shaped insect shows a kink-like behavior, which is consistent with the usual phase transition nature in the spin-liquid description[2]. The lattice size is 30×30 and the temperature is set to be $T = 4.3$ K from the experimental condition. The details are discussed in Supplementary Note 1.

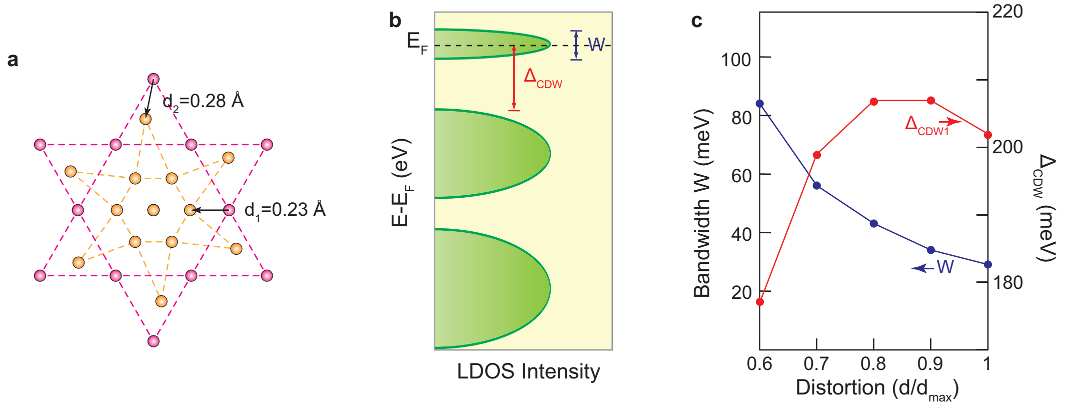

Supplementary Figure 3. **CDW-coupled bandwidth controlling.** **(a)** The atomic structure of the 1×1 triangular lattice (magenta dashed lines) and the David-star distortion (orange dashed lines). The atomic displacements are exaggerated. **(b)** The schematic view of the CDW-induced band splitting. The David star distortion makes the broad metallic band split into several subband manifolds with a narrow half-filled band at the Fermi level. There are two important energy scales to characterize the electronic structure of the CDW state, the bandwidth (W) of the narrow band and the CDW gap (Δ_{CDW}). The latter defined as the energy gap between the Fermi level and the edge of the lower subband reflects the extent of CDW order. **(c)** The evolution of the bandwidth and the CDW gap as a function of the degree of the David star distortion. We performed density-functional theory calculations that employ the generalized gradient approximation (GGA)[3] and the projector-augmented wave method[4], as implemented in VASP[5,6]. Valence electron wavefunctions were expanded in a plane wave basis set with a cutoff energy of 259 eV. The k-point integration was performed using a uniform with a 4×4 mesh for the Brilloin zone of the $(\sqrt{13} \times \sqrt{13})$ cell with the David-star distortion. The size of the distortion, using the linearly interpolated structures of the undistorted (1×1) and the fully relaxed $\sqrt{13} \times \sqrt{13}$ structure. At the full relaxation, W and Δ_{CDW} were calculated to be 30 meV and 202 meV, respectively. When the distortion diminishes, the bandwidth increases, while the CDW gap decreases, establishing a clear inverse proportion between them, in consistent with the earlier expectations[7,8]. The present result demonstrates that the CDW order can give us a extra controllability for the bandwidth-controlled metal-insulator transition in CDW-Mott insulator $1T$-TaS_2.

Supplementary Note 1 | Caculation details for a metal-insulator transition in a spin-liquid-type Mott insulator

The correlations between unpaired electrons on the center of David-star seem to be responsible for the electrically controlled metal-insulator transition. It can be described by an effective one-band Hubbard model on the triangular lattice at half filling

$$H = -t \sum_{\langle ij \rangle, \sigma} (c_{i\sigma}^{\dagger} c_{j\sigma} + H.c.) - \mu \sum_{i,\sigma} c_{i\sigma}^{\dagger} c_{i\sigma} + U \sum_{i} n_{i\uparrow} n_{i\downarrow}. \tag{1}$$

$c_{i,\sigma}^{\dagger}$ ($c_{i,\sigma}$) is an electron creation (annihilation) operator with spin σ at site i. The site i corresponds to the center of David-star. $n_{i,\sigma}$ is the number operator for spin σ at the site i. μ is an electron chemical potential, which fits the number of such unpaired electrons at half filling. U is the on-site Coulomb energy and t is the hopping integral between the nearest neighbors.

Resorting to the U(1) slave-rotor representation $c_{i\sigma} = e^{-i\theta_i} f_{i\sigma}$ for possible spin-liquid physics at least in the intermediate temperature regime[2], where bosonic field θ_i describes dynamics of collective charge fluctuations (sound modes) and $f_{i\sigma}$ expresses a fermionic field for spin degrees of freedom[9], we reconstruct an effective theory from the Hubbard model in terms of such bosonic and fermionic fields. As a result, it is given by

$$S_F = \int_0^{\beta} d\tau \left[\sum_{i,\sigma} f_{i\sigma}^{\dagger} (\partial_\tau - \mu) f_{i\sigma} - t\chi_f \sum_{\langle ij \rangle, \sigma} (f_{i\sigma}^{\dagger} f_{j\sigma} + H.c.) \right], \tag{2}$$

$$S_B = \int_0^{\beta} d\tau \left[\frac{1}{2U} \sum_{i} (\partial_\tau b_i^{\dagger})(\partial_\tau b_i) - t\chi_\theta \sum_{\langle ij \rangle} (b_{i\sigma}^{\dagger} b_{j\sigma} + H.c.) + \lambda \sum_{i} (|b_i|^2 - 1) + 2L^2 z t \chi_f \chi_\theta \right], \tag{3}$$

where the conventional saddle-point approximation has been performed for a spin-liquid-type Mott insulating phase. Here, χ_f and χ_θ describes band renormalization for electrons and the width of incoherent bands, respectively. λ is a Lagrange multiplier field to control the spin-liquid to Fermi-liquid phase transition, regarded as the chemical potential of bosons. These equations are based on a nonlinear σ-model description, where the rotor variable $e^{-i\theta_i}$ is replaced with b_i and unimodular constraint $|b_i|^2 = 1$ is taken into account[9]. $z = 6$ is the nearest coordinate number of the triangular lattice. L^2 is the size of system.

Performing the Fourier transformation and the Gaussian integration for both bosons and fermions, we obtain the mean-field free energy

$$F_{MF} = F_F + F_B + L^2 (2zt\chi_f \chi_\theta - \lambda), \tag{4}$$

$$F_F = -\frac{N_\sigma}{\beta} \sum_{\mathbf{k}} \ln \left[1 + e^{-\beta E_F(\mathbf{k})} \right], \tag{5}$$

$$F_B = \frac{1}{\beta}\sum_{\mathbf{k}}\left(\ln\left[1 - e^{-\beta E_B(\mathbf{k})}\right] + \ln\left[e^{\beta E_B(\mathbf{k})} - 1\right]\right), \tag{6}$$

where $E_F(\mathbf{k}) = t\chi_f\epsilon_{\mathbf{k}} - \mu$ is the dispersion of fermions and $E_B(\mathbf{k}) = \sqrt{2U(t\chi_\theta\epsilon_{\mathbf{k}} + \lambda)}$ is that of bosons. $N_\sigma = 2$ represents the spin degeneracy. $\epsilon_{\mathbf{k}}$ is the energy dispersion relation for electrons on the triangular lattice at $U = 0$ and $t = 1$.

Minimizing the effective free energy $F_{MF} = F_{MF}(\chi_f, \chi_\theta, \lambda)$ with respect to χ_f, χ_θ, and λ, we find a metal-insulator transition from a spin-liquid-type Mott insulator to a correlated metal at $U/t \approx 1.4$ as shown in Supplementary Figure 2b. It is important to note that the U and t can be controlled by the strength of the commensurate CDW ordering as discussed in the Supplementary Figure 3.

Considering the U(1) slave-rotor decomposition representation, it is straightforward to find that the electron spectral function is given by the convolution integral between fermion and boson propagators,

$$G(\mathbf{k}, i\omega) = \frac{1}{\beta}\sum_{i\Omega}\int\frac{d^2\mathbf{q}}{(2\pi)^2}G_F(\mathbf{k}+\mathbf{q}, i\omega + i\Omega)G_B(\mathbf{q}, i\Omega), \tag{7}$$

where

$$G_F(\mathbf{k}, i\omega) = \left[i\omega + \mu - t\chi_f\epsilon_{\mathbf{k}}\right]^{-1}, \quad G_B(\mathbf{k}, i\Omega) = \left[\frac{\Omega^2}{2U} + t\chi_\theta\epsilon_{\mathbf{k}} + \lambda\right]^{-1} \tag{8}$$

are fermion and boson propagators, respectively. Then, the electron spectral function consists of coherent and incoherent parts, given by

$$A_{\text{incoherent}}(\omega, \mathbf{k} = \mathbf{k}_F) = \int\frac{d^2\mathbf{q}}{(2\pi)^2}\frac{U}{E_B}\left[\{n_F(E_F) + n_B(E_B)\}\,\delta(\omega - E_F + E_B) - \{n_F(E_F) + n_B(-E_B)\}\,\delta(\omega - E_F - E_B)\right], \tag{9}$$

$$A_{\text{coherent}}(\omega, \mathbf{k} = \mathbf{k}_F) = N_0\delta(\omega) \tag{10}$$

at the Fermi energy. Here, $n_F(x)$ and $n_B(x)$ are Fermi-Dirac and Bose-Einstein distribution functions, respectively. $E_F = E_F(\mathbf{k}+\mathbf{q})$ and $E_B = E_B(\mathbf{q})$ are $\mathbf{q}$-dependent dispersions at $\mathbf{k} = \mathbf{k}_F$. N_0 is the condensation amplitude of bosons, which determines the height of the coherent peak in the correlated metallic phase ($N_0 \neq 0$).

As shown in Fig. 3f, an important result of the spin-liquid approach is that the coherent peak in the electron spectral function increases gradually with the decreasing U/t within the correlated metallic regime. And the incoherent part constitutes the double peaks identified with Hubbard bands. They turn out not only to exist inside the Mott insulating state but also to persist rather deep inside the correlated metallic state.

Supplementary References

---

[1] Stojchevska, L. *et al.* Ultrafast switching to a stable hidden quantum state in an electronic crystal. *Science* **344,** 177-180 (2014).

[2] Lee, S.-S., & Lee, P. A. U(1) Gauge Theory of the Hubbard Model: Spin Liquid States and Possible Application to κ-(BEDT-TTF)$_2$Cu$_2$(CN)$_3$. *Phys. Rev. Lett.* **95**, 036403 (2005).

[3] Perdew, J. P., Burke, K., & Ernzerhof, M. Generalized Gradient Approximation Made Simple. *Phys. Rev. Lett.* **77**, 3865 (1996).

[4] Blöchl, P. E. Projector augmented-wave method. *Phys. Rev. B* **50**, 17953 (1994).

[5] Kresse, G. and Furthmüller, J. Efficient iterative schemes for ab initio total-energy calculations using a plane-wave basis set. *Phys. Rev. B* **54**, 11169 (1996).

[6] Kresse, G. and Furthmüller, J. From ultrasoft pseudopotentials to the projector augmented-wave method. *Phys. Rev. B* **59**, 1758 (1999).

[7] Sipos, B. *et al.* From Mott state to superconductivity in 1T-TaS$_2$. *Nat. Mater.* **7,** 960-965 (2008).

[8] Perfetti, L. *et al.* Spectroscopic signatures of a bandwidth-controlled Mott transition at the surface of 1T-TaSe$_2$. *Phys. Rev. Lett.* **90,** 166401 (2003).

[9] Florens, S., & Georges, A. Slave-rotor mean-field theories of strongly correlated systems and the Mott transition in finite dimensions. *Phys. Rev. B* **70**, 035114 (2004).
